# Provider perspectives of implementation of an evidence-based insomnia treatment in Veterans Affairs (VA) primary care: barriers, existing strategies, and future directions

**DOI:** 10.1186/s43058-020-00096-4

**Published:** 2020-11-30

**Authors:** Erin Koffel, Hildi Hagedorn

**Affiliations:** 1 Center for Care Delivery and Outcomes Research, Minneapolis VA Health Care System, One Veterans Drive, Minneapolis, MN 55417 USA; 2 Department of Psychiatry, University of Minnesota Medical School, Minneapolis, MN USA

**Keywords:** Cognitive-behavioral therapy for insomnia (CBT-I), Sleep, Implementation, Dissemination, Qualitative research

## Abstract

**Background:**

Cognitive behavioral therapy for insomnia (CBT-I) is a highly effective nonpharmacological intervention that is widely considered the gold standard for insomnia treatment. Insomnia is a prevalent and debilitating public health concern. Up to one third of the general population struggles with chronic insomnia, greatly increasing the risk for chronic pain and inflammation, depression and suicide, and cognitive decline. Over the last 10 years, the Veterans Health Administration (VHA) evidence-based psychotherapy training program has trained nearly 1000 providers to deliver CBT-I in hospitals and clinics nationwide. Despite increased access, most patients with insomnia receive sleeping medications instead of CBT-I. This is particularly concerning for vulnerable populations, like older adults, who may be at increased risk of harms from medications. The goal of this study was to obtain a broad range of perspectives on CBT-I implementation from providers who commonly utilize and deliver CBT-I. This work identifies barriers and successful strategies used to overcome these barriers to guide future implementation efforts promoting evidence-based sleep care.

**Methods:**

Semi-structured interviews, using the Consolidated Framework for Implementation Research (CFIR) as a guide, were conducted with 17 providers from five Veterans Affairs (VA) facilities (8 primary care physicians, 4 primary care psychologists, and 5 CBT-I coordinators). We used a thematic analysis approach in which common ideas were identified across interviews and then grouped into larger conceptual themes. Data were concurrently collected and analyzed with rapid assessment process (RAP) techniques.

**Results:**

Findings suggested implementation barriers and facilitators related to the CFIR constructs of intervention characteristic (e.g., providers unfamiliar with primary evidence of CBT-I effectiveness), inner setting (e.g., sleep as a low relative priority in primary care), and outer setting (e.g., lack of external incentives for increasing CBT-I use), as well as several successful strategies, including use of local champions and supportive opinion leaders.

**Conclusions:**

These findings suggest promising opportunities to improve implementation of CBT-I, especially at facilities with less well-established CBT-I programs. Formal implementation trials are needed to systematically determine the real-world impact of strategies such as enlisting CBT-I champions, informing opinion leaders about CBT-I services, and promoting network weaving among primary care, mental health, and sleep clinics.

Contributions to the literature

- Primary care is an important target for improving insomnia care by increasing the use of cognitive-behavioral therapy for insomnia (CBT-I) using patient-, provider- and system-level implementation strategies.
- We found that future CBT-I programming efforts should identify and prepare local primary care champions and opinion leaders and promote network building across clinical teams to increase CBT-I use.
- These findings provide a roadmap for future research both within and outside of Veterans Affairs (VA) using implementation trials to systematically determine the real-world impact of various strategies, such as continuing education, expanding resources, and enlisting champions, especially at sites with less well-established CBT-I programs.

## Background

Insomnia is a debilitating, chronic condition that affects up to one third of the general population and involves difficulty falling or staying asleep and associated daytime dysfunction [1]. Sleep is crucial for physical and mental functioning, and extended periods of sleep disturbance have harmful downstream consequences, including increased risk for inflammation and chronic pain, higher rates of suicide, and dementia [2–5]. Identifying and treating insomnia is especially crucial in vulnerable subgroups at greater risk for sleep disturbance, including older adults, patients with physical and mental health comorbidities, and veterans [2, 6].

Cognitive behavioral therapy for insomnia (CBT-I) is recommended as the first-line treatment for insomnia by the American College of Physicians, the Veterans Affairs/Department of Defense (VA/DoD) insomnia treatment guidelines, and the American Academy of Sleep Medicine [7–9]. CBT-I is a nonpharmacological treatment that rapidly improves sleep quality by strengthening circadian rhythms (i.e., propensity to remain alert during the day and sleepy during night over a 24-h cycle), increasing the homeostatic sleep drive (i.e., increasing propensity for sleep through sustained and planned wakefulness), and alleviating insomnia-related anxiety. CBT-I has three defining elements: (1) sleep restriction, in which patients work with the provider to set a consistent bed-time and wake-time to eliminate time awake during the night, (2) stimulus control, in which patients get up if they cannot sleep and use the bed only for sleep, and (3) reduction of hyperarousal using cognitive therapy/mindfulness/relaxation. It is typically delivered in primary care or mental health specialty settings by a trained provider over approximately six sessions, either in-person or using telehealth delivery. Since 2010, the Veterans Health Administration (VHA) evidence-based psychotherapy training program has trained nearly 1000 providers to deliver CBT-I in hospitals and clinics nationwide [10]. As a highly scalable treatment, CBT-I has proven effectiveness in a variety of formats and is increasingly being delivered within VA clinics in convenient formats, including abbreviated sessions, telehealth, and online self-management [11–13].

Despite guideline consensus and increased access to CBT-I, most VA patients with insomnia receive sleeping medications instead of CBT-I, unnecessarily exposing them to risk of harms such as dementia, fractures, and driving impairment [14–16]. Previous work interviewing patients and providers has demonstrated system- and individual-level barriers to widespread use of CBT-I [17, 18]. The most common barriers include limited access to CBT-I providers, patient and provider lack of knowledge, and unsubstantiated beliefs about insomnia treatment (i.e., providers believe patients are not interested in sleep therapy, patients believe that the only thing doctors can offer are medications). However, existing studies are limited to single sites, specific provider groups, and study specific interview guides.

The current study provides a comprehensive investigation of CBT-I implementation progress and continued barriers across five VA facilities with long-standing, well-established CBT-I programs. Our goal was to obtain a broad range of perspectives on CBT-I implementation progress and barriers by including providers who commonly refer patients to CBT-I (i.e., primary care physicians) and providers who deliver CBT-I (i.e., psychologists), as well as CBT-I coordinators who have been actively involved in dissemination efforts. We focused on providers since they are crucial to the success of wide-scale implementation of evidence-based treatments and provide an important and understudied counterpart to patient perspectives on CBT-I. We used the Consolidated Framework for Implementation Research (CFIR) to systematically evaluate the implementation context and progress of CBT-I in VA and to ensure that findings would be broadly applicable both within and outside of VA [19]. CFIR is an organizing framework consisting of five major domains: intervention characteristics, outer setting, inner setting, characteristics of the individuals involved, and the process of implementation [19]. It has been recommended as a unifying structure for gathering data on the major constructs that influence implementation of evidence-based treatments [20].

## Methods

### Overall design

We conducted individual semi-structured interviews with 17 VA providers. The local institutional review board approved all procedures, and we obtained verbal informed consent from all participants prior to conducting interviews. The current study used CFIR as a framework to provide a comprehensive evaluation of constructs relevant for CBT-I implementation within VA. The main goal of the study was to identify barriers and successful strategies used to overcome these barriers to guide future implementation efforts and promote the gold-standard of insomnia care for patients struggling to sleep.

### Participants

Participants were 17 VA providers. Providers included 8 primary care physicians, 4 primary care mental health integration (PCMHI) psychologists, and 5 CBT-I coordinators from five VA facilities nationwide. PCMHI providers within VA deliver brief behavioral therapy within primary care, with an emphasis on same-day access. An average of 3 providers were interviewed at each facility, ranging from 2 to 5 providers per facility. Providers were mainly located in primary care where most insomnia care is provided. The included facilities had well-established CBT-I programs (i.e., experienced CBT-I coordinator managing active clinician group) and were selected for maximal geographic representation, including east and west coast, Midwest, and southeastern states.

Invitation emails were sent to potentially eligible providers (*n* = 28), followed by phone interviews if providers expressed interest in participating (*n* = 17). Starting with CBT-I coordinators, a snowball sampling approach was used whereby participants were asked to recommend additional providers who might be interested in participating. Recruitment and enrollment continued until thematic saturation was achieved and further interviews did not provide new or additional substantial information. As discussed below, we analyzed data concurrently with data collection in order to saturate each domain captured by the interview guide for each facility and provider type.

### Data collection

Interviews were conducted over telephone by the first author, a clinical psychologist with qualitative research training and experience. Interviews took place at VA facilities, lasted 15–30 min, and were recorded directly to a secure VA server with participants’ verbal consent. Questions in the semi-structured interview guide were chosen based on CFIR recommended constructs for investigating implementation of evidence-based treatments and prior research on CBT-I dissemination [17, 19] (see appendix). Providers were asked about their familiarity with and use of CBT-I, awareness of the evidence for CBT-I effectiveness, screening and referral process for CBT-I, CBT-I resources available in primary care, perspectives on different modes of CBT-I delivery (including telehealth, online course, electronic applications), relative priority of CBT-I, experience with patient feedback about CBT-I, and process for finding out new information about CBT-I resources and initiatives.

### Data analysis

We used a thematic analysis approach in which common ideas were identified across interviews and then grouped into larger conceptual themes [21]. We employed rapid assessment process (RAP) techniques to analyze the data, using concurrent data collection and analysis [22, 23]. RAP techniques have been recommended as a way of quickly analyzing time sensitive qualitative data and are commonly utilized in health services research to develop and refine health interventions [22, 23]. This approach involves creating and refining a summary template that includes all the domains captured by the interview guide. Interviews are then summarized using the template. Summary points are transferred to a matrix to facilitate synthesis of important findings. In this study, a summary template was developed that included all the domains captured by the interview guide; these domains included CFIR constructs, as well as any additional domains that emerged during interviews. This template was completed for all interviews based on review of recordings and interviewer notes. Summary points from the templates were transferred to a matrix, grouped by facilities and provider type, to facilitate synthesis of core themes and concepts [24]. The second author, a clinical psychologist and expert qualitative researcher who was not involved in conducting the interviews, reviewed the matrix. Both authors independently generated an initial set of cross-cutting themes based on matrix review and then met for discussion and consensus on final higher order themes.

## Results

**Fig. 1:**
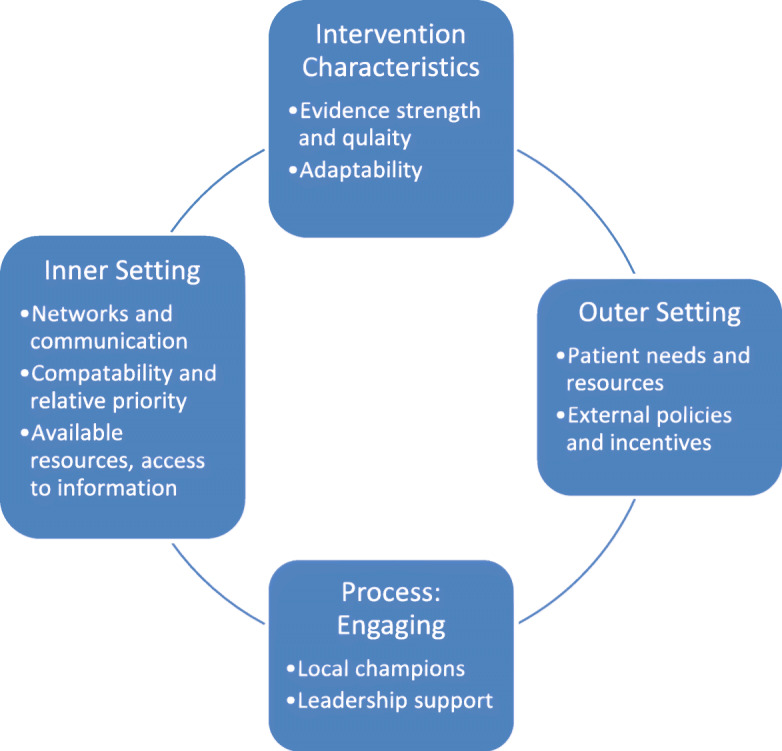
Barriers and facilitators related to implementation of CBT-I in VA

Interviews identified implementation barriers and facilitators for CBT-I use in primary care. These barriers and facilitators fell under three broad CFIR constructs: intervention characteristics, inner setting, and outer setting. In addition, interviews revealed the importance of the implementation process of engaging, including active CBT-I champions and strong support from opinion leaders. These barriers and facilitators are described in detail below and outlined in Fig. 1.

### Intervention characteristics

#### Evidence strength and quality

All primary care physicians interviewed were familiar with CBT-I as an effective treatment for insomnia offered within VA, but most were unfamiliar with specific guidelines or primary evidence. Knowledge of CBT-I was mainly derived from local champions and on-the-job training rather than systematic education. As one physician said:

I have been educated in research talks from an expert in the area. I’ve forgotten the specifics of studies that show effectiveness, but I believe it is effective based on what I have heard.

Some physicians utilized CBT-I as a first-line treatment and had a “low-threshold” for making referrals, whereas others preferred to start with sleep hygiene advice and medication, making a CBT-I referral later if insomnia persisted. One PCMHI psychologist suggested integrating CBT-I education into resident lectures about general primary care problems, like pain, anxiety, and depression to increase the use of CBT-I as a first-line treatment.

Primary care physicians noted that positive feedback from patients is influential in increasing CBT-I referrals and “getting doctors on board.” Reviews from patients were mixed, with some enthusiastically reporting improved sleep after decades of insomnia and others reporting no treatment effect. One physician used positive testimonials from past patients to sell the treatment to new patients. Physicians acknowledged that negative reviews may reflect a lack of engagement rather than a failure to benefit from CBT-I.

#### Adaptability

All providers appreciated the potential scalability and convenience of CBT-I for primary care settings, including brief versions of the treatment and telehealth delivery. Self-management CBT-I, in which patients independently complete an online web-course or paper-based treatment manual, received mixed reviews. PCMHI psychologists were the most positive about self-management and felt that this fit within their model of care and increased access, especially if it was combined with provider check-ins. Primary care physicians and CBT-I coordinators were concerned about patient follow-through and comorbid sleep disorders, such as sleep apnea, that may not be diagnosed and treated with a self-management approach. One physician preferred in-person CBT-I to self-management because it provides a “foot in the door” for patients that need additional mental health treatment:

I like sending them out to PCMHI because I secretly hope they will address some of their other underlying mental health issues so sometimes I use CBT-I as a foot in the door. I’ve had a couple patients where sleep is a major issue, but so are these other things that they are not ready to address yet, so I hope that once they start talking, they’ll keep talking.

In general, primary care and CBT-I providers were more likely to consider self-management for patients without comorbidities and as a last resort for those who were unable or unwilling to work with a CBT-I provider. Primary care physicians also felt they would not have enough information or time to provide patients with self-management resources and training.

### Inner setting

#### Networks and communication

*S*trong connections between primary care clinics, PCMHI teams, and sleep medicine clinics increased utilization of CBT-I. Within VA, CBT-I training is provided through the VA Office of Mental Health and Suicide Prevention (OMHSP) and many CBT-I providers are located within mental health clinics. Highly successful CBT-I programs were often closely integrated with both mental health and sleep medicine clinics and functioned as a sleep team or community, with shared consult and referral systems. As one CBT-I coordinator said:

A patient is typically sent the sleep disorders clinic. […] The consult is reviewed by one of our sleep fellow and there is an internal consult that goes to the behavioral sleep medicine clinic and they get CBT-I.

These sleep teams provided ongoing services and education within primary care clinics. In some cases, individual relationships increased CBT-I use. For example, primary care residents who were exposed to CBT-I champions during their training often took full time staff positions and went on to utilize CBT-I.

#### Compatibility and relative priority

Primary care physicians did not proactively assess for insomnia and sleep loss was not considered as urgent as other health concerns. As one physician said:

If someone convinced me that there was a strong causal pathway between inadequate sleep and serious morbidity and mortality, then I would be thinking ‘wow we need to move on this asymptomatic killer of people.’ It is hard for me to think of insomnia as one of those asymptomatic killers we are supposed to screen for.

Widespread screening for insomnia was not seen as compatible with primary care appointments given limited time, competing demands of other medical conditions, and patient costs (e.g., patient fatigue, damage to rapport if patients get annoyed with screening). There was also an assumption that mental health providers were assessing and treating insomnia. Physicians suggested that lack of screening may also be related to uncertainty about how to treat insomnia, a preference for not prescribing sleeping medication and a general reluctance to bring up sleep because of a preference for not prescribing medication. The general consensus among physicians was that sleep took a “backseat priority” in complex patients and was not prioritized by patients or providers. As one physician said:

Sleep is definitely a backseat priority. It’s important and it affects everything but patients are coming in with blood pressure at 170 over 100 and their A1C is 12 and they are sobbing because of their…..whatever. And patients don’t always recognize how much sleep affects them and you have to get patients to buy in especially if it involves behavioral change. And they just want a pill, they want me to prescribe Zolpidem because they perceive that does help and they don’t want to do the hard work.

Both PCMHI psychologists and CBT-I coordinators expressed the need for more provider education about the importance of sleep, but also acknowledged that providers are stuck between wanting to decrease sleeping medication and their patients not wanting to engage in CBT-I.

If patients brought up insomnia during appointments, primary care physicians commonly referred them for further evaluation and treatment. Physicians were comfortable providing a basic overview of CBT-I to their patients but did not have the knowledge or time to describe the treatment in-depth. Several physicians wanted written guidance on how to describe CBT-I to patients. CBT-I coordinators pointed out that comprehensive sleep evaluation and treatment discussion is part of the CBT-I intake; however, it may be helpful to have primary care providers set the stage:

[Patients] have no clue why a behavioral intervention is related to insomnia at all. The backdrop is you treat insomnia with sleep medications so what in the world could I do sitting in a room talking with people, how would that help my sleep. They can’t even wrap their head around it. So the primary care provider has to be the salesperson for that intervention. A good chunk of people don’t show up or engage because they don’t see how it applies to them.

In contrast to primary care physicians, PCMHI psychologists viewed insomnia screening and treatment as highly compatible with their model of care. They routinely screened for insomnia during functional assessments and prioritized sleep treatment in accordance with patients’ treatment goals. PCMHI psychologists found CBT-I easy to deliver in a PCMHI setting, especially in briefer formats.

#### Available resources and access to information and knowledge

Most primary care physicians were satisfied with CBT-I resources in their facility. They reported having ready access to CBT-I providers through electronic medical record consults and warm-hand offs to PCMHI teams (i.e., patient has same day appointment with PCMHI team). A variety of consults were used across and within facilities, including general sleep medicine, behavioral sleep medicine, mental health, and PCMHI, and this led in some cases to confusion about how to refer to CBT-I. Several primary care physicians expressed the desire for a more centralized resource to learn about CBT-I and make referrals, something easy and “within their pattern of practice” such as established order sets within the electronic medical record. This was particularly useful after-hours or on weekends when warm-hand offs were more difficult. For example, one primary care physician said:

There’s no central repository of information, no place you can go for a reference.[…] Sometimes you just want to find something out without going to track someone down.

In contrast to primary care physicians, PCMHI psychologists and CBT-I coordinators expressed a need for more CBT-I providers within primary care, especially in off-site clinics. Demand often overwhelmed supply, with up to 6 months wait for primary care patients to see a CBT-I provider. One PCMHI provider suggested the need for local training due to the limited number of slots within the national training program. Telehealth was suggested as a potential way of increasing access within PCMHI settings.

### Outer setting

#### Patient needs and resources

There was a consensus that CBT-I provides a valuable nonpharmacological treatment option for patients struggling with insomnia. There were contrasting views among primary care physicians about patient willingness to consider CBT-I. One physician noted that patients were generally receptive since “not many patients just want medications.” Conversely, another estimated that up to half of his patients were not interested in CBT-I since they “just want trazodone and to take care of it all in primary care.” One CBT-I coordinator emphasized the need to educate both physicians and patients about the availability of nonpharmacological options like CBT-I:

My experience is providers are really uncomfortable with meds but they perceive that is what their patients want. […] They have this tension. We asked patients why they didn’t talk to their doctor and they said, ‘all my doctor has to give me are pills and why would I bring it up if I didn’t want one.’ And we talked to providers who said, ‘my patients come asking for pills and I don’t know what else to do.’

Nearly all physicians downplayed the mental health aspects of CBT-I when making referrals, avoiding the terms “psychologist” and “therapy.” They felt patients were more receptive when CBT-I was described as a “sleep intervention” delivered by “sleep experts.” Several physicians commented that the behavior change is more difficult than taking sleeping medication and that some patients do not understand how sleep affects health. They recommended patient education to promote wide-spread uptake of CBT-I.

#### External policies and incentives

One primary care physician pointed out that it was difficult to focus on increasing the use of behavioral sleep treatments as an alternative to sleeping medication because there was no measurable data to show improvement in practice, unlike other deprescribing initiatives:

Sleep doesn’t have measurable data where you can say we reduced our benzo users from 20% to 10%. You can’t say I fixed insomnia [treatment] by 10%. A lot of things we follow have measurable action items.

A CBT-I coordinator suggested that efforts to increase the uptake of CBT-I be linked to deprescribing initiatives, with CBT-I offered as an alternative to initiating or continuing sedative hypnotic prescriptions, including benzodiazepines. Several primary care physicians reported that they are already taking this approach. As one said:

I’m in a long-term project with my folks who have been on sleeping pills to change the way we are managing their sleep.

Similarly, CBT-I coordinators are advertising CBT-I as a safer and more effective alternative to medications to increase referrals:

It helps that the DoD/VA guidelines came out. […] I’ve been waving that like a big flag and using that to highlight that CBT-I is best practice. No one is saying that medication is better.

### Importance of engaging

Providers identified two key facilitators that they believed contributed to successful implementation and widespread dissemination of CBT-I: local champions and leadership support. Local champions represented the most consistent and effective method of increasing insomnia awareness and CBT-I referrals within primary care. Primary care physicians who routinely utilized CBT-I were made aware of resources through formal talks and informal conversations with local champions. As a result of these contact, they were more likely to be listening for sleep problems among their patients and have a “lower bar” for CBT-I referrals.

CBT-I coordinators at well-established CBT-I programs suggested that leadership support was crucial in growing their services. They described how CBT-I was “warmly embraced” with “a lot of enthusiasm” by leadership in sleep medicine, primary care, and mental health. Leadership support took the form of educational initiatives, rolling out CBT-I clinics and protected time for CBT-I providers. Supportive leaders valued CBT-I as an essential service and these attitudes had a trickle-down effect to primary care clinics, increasing CBT-I referrals. One CBT-I coordinator described the positive reception for CBT-I programming at a primary care meeting:

Primary care providers here overall prefer not to write prescriptions for sleep. […] Someone said ‘we hear you are developing a non-medication treatment program for insomnia’ and he said ‘yes we are’ and he actually got a round of applause because they were so happy to have an option that wasn’t sleeping medication.

It was noted that receptiveness to CBT-I varies substantially among sites and that lack of leadership support has a chilling effect on referrals and provider willingness to engage.

## Discussion

The current study provides a broad range of provider perspectives on CBT-I implementation progress and continued barriers across five VA facilities with long-standing, well-established CBT-I programs. Findings suggested implementation barriers and facilitators related to the CFIR constructs of intervention characteristic (e.g., providers unfamiliar with primary evidence of CBT-I effectiveness), inner setting (e.g., sleep as a low relative priority in primary care), and outer setting (e.g., lack of external incentives for increasing CBT-I use), as well as several successful strategies, including use of local champions and supportive opinion leaders. This replicates previous work identifying barriers related to provider knowledge and beliefs [17, 18] and extends this work to include previously unexplored barriers like compatibility and relative priority and external policies and incentives.

### Implications

Continuing to expand the reach of CBT-I in primary care will require thoughtful and systematic testing of patient-, provider-, and organizational-level implementation strategies. We review several evidence-based strategies from the Expert Recommendations for Implementing Change (ERIC) project that address the main CFIR barriers identified in the interviews [25, 26].

#### Continuing education

There has been substantial progress over the last 10 years in educating providers about the importance of sleep and the use of CBT-I as a first-line treatment for insomnia [7, 8, 10]. However, most providers still do not actively assess and manage sleep problems. This may be due to several reasons, including not perceiving sleep loss as important, diffusion of responsibility (i.e., assuming insomnia is treated in mental health), uncertainty about treatment options, and the desire to avoid prescribing sedative-hypnotics. Some providers default to sleep hygiene and medication before referring patients to CBT-I.

It will be important to continue disseminating evidence and practice guidelines using educational meetings and materials. Provider education about the difference between sleep hygiene and CBT-I and how to describe CBT-I to patients is needed. Involving local champions and patients would lend credibility to these efforts. For example, patient testimonials and preferences for non-medication treatment options could be integrated into educational materials to get more providers “on-board.” Local consensus discussions may be helpful in addressing whether increasing the uptake of CBT-I for insomnia in primary care is important and appropriate within each facility. Widespread insomnia screening may not be a good fit in primary care given time constraints and competing demands, but continued education can help put sleep on the radar for primary care physicians and increase CBT-I referrals. It is important to note that assessing insomnia severity and screening for comorbid sleep disorders is essential for appropriate sleep care, but this role may be better served by CBT-I providers. Finally, integrating CBT-I into ongoing deprescribing initiatives was also recommended to motivate providers to use CBT-I.

Interviews revealed a need to educate and motivate patients about the health effects of poor sleep, the risks of long-term medication use for insomnia, and the long-term benefit of CBT-I, as well as preparing patients to be active participants in their insomnia care. Many providers perceived patients to be unready for the behavior change required in CBT-I and looking for a quick fix through sleeping medications (i.e., in their experience, some patients did not follow through with referrals to CBT-I). Future implementation efforts may want to consider direct-to-patient educational materials to provide motivation and education, such as brochures mailed to patients’ homes or available in primary care clinics. Providers recommended telling patients about CBT-I using the context of sleep health rather than mental health to increase patient willingness to engage.

#### Expanding resources

CBT-I is valued as a scalable treatment that can be delivered in many formats and modalities. More CBT-I providers are needed in primary care to deliver CBT-I using brief formats or telehealth. As the menu of CBT-I treatment options increases, it will be important to keep providers informed about available treatments. Providers recommended a centralized referral source that fits within current practice patterns to make accessing CBT-I resources and referrals fast and easy. It will be equally important to talk to providers about feasibility and acceptability of new forms of CBT-I delivery to make sure they will be used in real-world clinical practice. For example, providers had concerns about self-management CBT-I resources that would need to be addressed for widespread use.

#### Enlisting champions, informing opinion leaders, and promoting network weaving

Enthusiastic champions were one of the strongest facilitating factors. Champions increased CBT-I knowledge and utilization through individual relationships and wide-spread educational efforts. For example, CBT-I champions at the facilities interviewed gave regular talks about CBT-I during existing clinic meetings, personally discussed CBT-I referrals with individual providers, and actively promoted CBT-I among their colleagues in primary care and sleep medicine. Future implementation work could focus on identifying and preparing local primary care champions as part of national CBT-I training effort. These individuals could market CBT-I to providers and support efforts to increase referrals at their facilities.

In addition to champions, leadership support provided the resources and credibility needed to increase CBT-I referrals. This included supporting educational initiatives and CBT-I clinics, as well as protected time for CBT-I providers. Future implementation work could focus on identifying and informing local opinion leaders about CBT-I with the hope that they would promote this treatment among colleagues. Finally, strong CBT-I sites also had networks and relationships that helped promote CBT-I, especially among trainees who may later become part of organization. Interviews revealed the importance of a “sleep partner” either through PCMHI or sleep medicine clinics who can “suss out” sleep problems and provide treatment. Implementation efforts should promote network weaving across primary care, mental health, and sleep medicine to encourage shared information, problem-solving, and the shared goal of improving insomnia care by increasing CBT-I.

### Limitations and future directions

The general recommendations reviewed above are made with the understanding that there are varying degrees of progress with implementing CBT-I among facilities. This study interviewed providers at VA facilities with well-established CBT-I programs and may not be representative of the views of providers at other facilities. Additional work will be needed to determine if sites earlier in the CBT-I implementation process have additional or different barriers/facilitators.

The snowball sampling approach also introduced the potential for sampling bias. For instance, recommended providers may be more familiar with or view CBT-I in a more positive light than other providers who were not recommended to participate in this study. In addition, findings may not generalize to all patients, including special groups such as women veterans. It is important to note that we did not obtain patient perspectives on implementation barriers in the current study, although our previous work has included interviews with patients about barriers to CBT-I referrals and use [18]. Future research may benefit from a comprehensive exploration of CFIR constructs from patients’ point of view, with a focus on key segments of the patient population like older adults or women veterans. Despite these limitations, findings have relevance within and outside of VA as a growing number of guidelines recommend CBT-I as a first-line treatment for insomnia.

## Conclusion

The goal of these interviews was to capture and share local knowledge from successful implementation sites on what has worked (and not worked) in promoting CBT-I in primary care. Future work will most likely benefit from local needs assessment and tailoring of strategies to the needs of specific facilities. In addition, formal implementation trials are needed to systematically determine the real-world impact of various strategies. Although much work remains to be done, our hope is that the lessons learned over the last 10 years of implementing CBT-I within VA provides valuable groundwork to promote the spread of CBT-I for the millions of patients who need safe and effective insomnia care.

## Supplementary Information

**Additional file 1.** Appendix Review Guide

## Data Availability

The datasets analyzed during the current study are available from the corresponding author on reasonable request. These data however will remain within the VA firewall and be housed on VINCI data servers. Outside investigators can follow VA procedures and receive training and approval for access within VA firewalls.

## Abbreviations

CBT-I: Cognitive behavioral therapy for insomnia
CFIR: Consolidated Framework for Implementation Research
ERIC: Expert Recommendations for Implementing Change
VA/DoD: Veterans Affairs/Department of Defense
VHA: Veterans Health Administration

## References

[1] Roth T (2007). Insomnia: definition, prevalence, etiology, and consequences. J Clin Sleep Med.

[2] Koffel E, McCurry SM, Smith MT, Vitiello MV (2019). Improving pain and sleep outcomes in middle-aged and older adults: the promise of behavioral sleep interventions. Pain..

[3] McCarthy MS, Hoffmire C, Brenner LA, Nazem S. Sleep and timing of death by suicide among U.S. Veterans 2006-2015: analysis of the American Time Use Survey and the National Violent Death Reporting System. Sleep. 2019;42(8). 10.1093/sleep/zsz094 31180507

[4] Vargas I, Perlis ML, Grandner M, Gencarelli A, Khader W, Zandberg LJ, et al. Insomnia symptoms and suicide-related ideation in U.S. Army Service Members. Behav Sleep Med. 2019:1-17. 10.1080/15402002.2019.1693373 31738588

[5] Bubu OM, Brannick M, Mortimer J, Umasabor-Bubu O, Sebastiao YV, Wen Y, et al. Sleep, Cognitive impairment, and Alzheimer’s disease: a systematic review and meta-analysis. Sleep. 2017;40(1). 10.1093/sleep/zsw032 28364458

[6] Jenkins MM, Colvonen PJ, Norman SB, Afari N, Allard CB, Drummond SP (2015). Prevalence and mental health correlates of insomnia in first-encounter veterans with and without military sexual trauma. Sleep..

[7] Qaseem A, Kansagara D, Forciea MA, Cooke M, Denberg TD (2016). Management of chronic insomnia disorder in adults: a clinical practice guideline from the American College of Physicians. Ann Intern Med.

[8] Department of Veterans Affairs, Department of Defense. VA/DoD clinical practice guidelines for the management of chronic insomnia disorder and obstructive sleep apnea [Internet]. (2019) {cited 2019 Apr 16}.

[9] Schutte-Rodin S, Broch L, Buysse D, Dorsey C, Sateia M (2008). Clinical guideline for the evaluation and management of chronic insomnia in adults. J Clin Sleep Med.

[10] Manber R, Carney C, Edinger J, Epstein D, Friedman J, Haynes PL (2012). Dissemination of CBTI to the non-sleep specialist: protocol development and training issues. J Clin Sleep Med.

[11] Bramoweth AD, Germain A, Youk AO, Rodriguez KL, Chinman MJ. A hybrid type I trial to increase Veterans’ access to insomnia care: study protocol for a randomized controlled trial. Trials. 2018. 10.1186/s13063-017-2437-y PMC5787269 29373993

[12] Koffel E, Kuhn E, Petsoulis N, Erbes CR, Anders S, Hoffman JE (2018). A randomized controlled pilot study of CBT-I coach: feasibility, acceptability, and potential impact of a mobile phone application for patients in cognitive behavioral therapy for insomnia. Health Informatics Journal.

[13] Sarmiento KF, Folmer RL, Stepnowsky CJ, Whooley MA, Boudreau EA, Kuna ST (2019). National expansion of sleep telemedicine for veterans: the TeleSleep program. J Clin Sleep Med.

[14] Ulmer CS, Bosworth HB, Beckham JC, Germain A, Jeffreys AS, Edelman D (2017). Veterans affairs primary care provider perceptions of insomnia treatment. J Clin Sleep Med.

[15] Shayegani R, Song K, Amuan ME, Jaramillo CA, Eapen BC, Pugh MJ (2018). Patterns of zolpidem use among Iraq and Afghanistan veterans: a retrospective cohort analysis. PLoS One.

[16] Wilt T, MacDonald R, Brasure M, Olson CM, Carlyle M, Fuchs E (2016). Pharmacological treatment of insomnia disorder: an evidence report for a clinical practice guideline by the American College of Physicians. Ann Intern Med.

[17] Koffel E, Bramoweth AD, Ulmer CS (2018). Increasing access to and utilization of cognitive behavioral therapy for insomnia (CBT-I): a narrative review. J Gen Intern Med.

[18] Koffel E, Amundson E, Polusny G, Wisdom J. “You’re missing out on something great”: patient and provider perspectives on increasing the use of cognitive behavioral therapy for insomnia. Behav Sleep Med. 2019. 10.1080/15402002.2019.1591958 PMC6759412 30907144

[19] Damschroder LJ, Aron DC, Keith RE, Kirsh SR, Alexander JA, Lowery JC (2009). Fostering implementation of health services research findings into practice: a consolidated framework for advancing implementation science. Implement Sci.

[20] Damschroder LJ, Hagedorn HJ (2011). A guiding framework and approach for implementation research in substance use disorders treatment. Psychol Addict Behav.

[21] Miles MB, Huberman AM, Saldaña J (2013). Qualitative data analysis: a methods sourcebook.

[22] Beebe J (2001). Rapid assessment process: an introduction.

[23] McMullen CK, Ash JS, Sittig DF, Bunce A, Guappone K, Dykstra R (2011). Rapid assessment of clinical information systems in the healthcare setting: an efficient method for time-pressed evaluation. Methods Inf Med.

[24] Averill JB (2002). Matrix analysis as a complementary analytic strategy in qualitative inquiry. Qual Health Res.

[25] Powell BJ, Waltz TJ, Chinman MJ, Damschroder LJ, Smith JL, Matthieu MM (2015). A refined compilation of implementation strategies: results from the expert recommendations for implementing change (ERIC) project. Implement Sci.

[26] Waltz TJ, Powell BJ, Fernandez ME, Abadie B, Damschroder LJ (2019). Choosing implementation strategies to address contextual barriers: diversity in recommendations and future directions. Implement Sci.

