# Supplementary material for: Provider perspectives of implementation of an evidence-based insomnia treatment in Veterans Affairs (VA) primary care: barriers, existing strategies, and future directions
Source: Implement Sci Commun. 2020 Nov 30;1:107. doi: 10.1186/s43058-020-00096-4 (PMC7706055; doi:10.1186/s43058-020-00096-4)
Supplement: Supplementary file 1 — Additional file 1. Appendix Review Guide [file 43058_2020_96_MOESM1_ESM.docx]

Interview Guide

1. Are you familiar with Cognitive Behavioral Therapy for Insomnia (CBT-I)? (if not, describe)
2. What kind of information or evidence are you aware of that shows whether or not CBT-I will work in primary care?
   - What evidence have you heard about from your own research? Practice guidelines? Published literature? Co-workers? Other settings?
   - What kind of supporting evidence or proof is needed to get staff on board?
3. What is the best way for patients to access CBT-I? How would you communicate to patients about this intervention?
   - What types of patients would you refer to CBT-I? Do you screen for insomnia?
4. Is there a need for more CBT-I resources in primary care?
   - What resources would be helpful for providers?
   - What kinds of changes or alterations do you think we could make to the CBT-I treatment program so it will work effectively in your setting?
5. What do you think about self-management CBT-I? (provide examples)
   - What are likely issues or complications that may arise?
   - What advantages does self-management have compared to existing programs?
   - What disadvantages does the intervention have compared to existing programs?
   - What types of patients would you refer to self-management vs. standard provider delivered CBT-I? In what ways will the intervention meet their needs?
6. To what extent might CBT-I take a backseat to other high-priority initiatives going on now? To what extent might sleep treatment take a backseat to other conditions?
   - How important do you think it is to provide CBT-I resources compared to the other priorities?
7. Have you heard stories about the experiences of patients with CBT-I?
   - Can you describe a specific story?
8. How do you typically find out about new information, such as new initiatives, accomplishments, issues, new staff, staff departures? (staff meetings, talking to people informally) Who are your go to people for questions about behavioral insomnia treatment? How available are these individuals
9. How prepared are you to use CBT-I in your current practice?
